# Supplementary material for: Dynamic transcriptomic profiles of zebrafish gills in response to zinc supplementation
Source: BMC Genomics. 2010 Oct 11;11:553. doi: 10.1186/1471-2164-11-553 (PMC3091702; doi:10.1186/1471-2164-11-553)
Supplement: Additional file 2 — Interactive Direct Interaction Network representing the molecular interactions between zinc, copper, iron, calcium and proteins encoded by transcripts changed by zinc supplementation. Mini web-site containing index.html and hyperlinked pages in subdirectory describing a Direct Interaction Network automatically generated based on curated interactions contained within the proprietary PathwayArchitect database. Ovals represent proteins and the circles symbolize metal ions. Objects are coloured by their abundance in zebrafish at the time-point they were significantly different from the control is a scale from -4 fold (dark green) to +4 fold (dark red). Where significant differences were found at more than one time-point, the colour overlay shows expression at the first instance. Dark blue squares denote 'binding', and light blue squares 'expression'; green squares stand for 'regulation', green diamonds for 'metabolism', and green circles for 'promoter binding'. Arrow heads indicate directionality of the interaction where annotated. All nodes and edges can be further interrogated by selecting the relative area of the image. [file 1471-2164-11-553-S2.zip › PathwayArchitect Zn xs DIN/637549.html]

# REGULATION:

|  |  |
| --- | --- |
| Type | REGULATION |
| Effect | Negative |


---

|  |  |
| --- | --- |
| Score | 0 |


---

|  |  |
| --- | --- |
| Reference Count | 8 |


---

|  |  |
| --- | --- |
| Mechanism | Unknown |


---

|  |  |
| --- | --- |
| Reference:0 || Sentence | "However, dietary Fe deficiency increased the hepatic Zn in response to Ni or Cd and the hepatic Ca in response to Pb administration." |
| PMID | 8029692 |
| Year | 1994 |
| Species | Rat |
| Journal | Sci Total Environ |
| RefScore | 0 |
| Source | PArchNLP |
  |
|  |  |


---

|  |  |
| --- | --- |
 Reference:1 || Sentence | "Thus, the possibility that intrinsic iron in formulas for feeding infants and in vitamin-mineral supplement might inhibit the absorption of zinc justifies concern about the Fe/Zn ratio in the formulation of these products." |
| PMID | 7223699 |
| Year | 1981 |
| Species | Human |
| Journal | Am J Clin Nutr |
| RefScore | 2 |
| Source | PArchNLP |
  ||  |  |


---

|  |  |
| --- | --- |
 Reference:2 || Sentence | "Twelve JCA patients had reduced serum iron (Fe), 6 reduced serum zinc (SZn), 14 reduced intra-erythrocytic zinc (EZn) and 2 reduced serum copper (SCu)." |
| PMID | 8652951 |
| Year | 1995 |
| Species | Human |
| Journal | Acta Paediatr |
| RefScore | 1 |
| Source | PArchNLP |
  ||  |  |


---

|  |  |
| --- | --- |
 Reference:3 || Sentence | "Iron supplementation caused no additional increase in the TBARS in hyperthyroid rats, ameliorated the decrease in GSH content and abolished the induction of Cu, Zn SOD." |
| PMID | 15227727 |
| Year | 2004 |
| Species | Human |
|  | Rat |
| Journal | Yonsei Med J |
| RefScore | 1 |
| Source | PArchNLP |
  ||  |  |


---

|  |  |
| --- | --- |
 Reference:4 || Sentence | "Consideration of solutions to these problems, including conscious adjustment of the Fe/Zn ratios in human diets, foods and therapeutic nutrient supplements in order to reduce the zinc-inhibiting effects of iron, should become a priority in policy and marketing discussions within government regulatory agencies, industry and the scientific community of human and clinical nutritionists." |
| PMID | 3522825 |
| Year | 1986 |
| Species | Human |
| Journal | J Nutr |
| RefScore | 0 |
| Source | PArchNLP |
  ||  |  |


---

|  |  |
| --- | --- |
 Reference:5 || Sentence | Furthermore, there was a significant effect of iron deficiency (P < 0.05) on increasing zinc concentration and a statistical trend (P = 0.08) toward iron deficiency-induced copper accumulation in the globus pallidus. |
| Year | 2004 |
| PMID | 15157939 |
| Species | Rat |
| Journal | J Nutr Biochem |
| RefScore | 1 |
| Source | PArchNLP |
  ||  |  |


---

|  |  |
| --- | --- |
 Reference:6 || Sentence | Previous studies have demonstrated increased serum copper and iron levels and decreased selenium and zinc levels in patients with myocardial infarction. |
| PMID | 15966572 |
| Year | 2005 |
| Species | Human |
| Journal | J Trace Elem Med Biol |
| RefScore | 2 |
| Source | PArchNLP |
  ||  |  |


---

|  |  |
| --- | --- |
 Reference:7 || Sentence | Zinc depletion produced mild-moderate iron deficiency in addition to zinc deficiency, which could be corrected by repletion with iron and zinc. |
| PMID | 16198227 |
| Year | 2005 |
| Species | Rat |
| Journal | Free Radic Biol Med |
| RefScore | 3 |
| Source | PArchNLP |
  |


---

|  |  |
| --- | --- |
